# In Situ Polyimide Modified Polymethylsilsesquioxane Aerogels for Thermal Insulation

**DOI:** 10.3390/gels12100898

**Published:** 2026-10-04

**Authors:** Ziqi Zhu, Junyong Chen, Defang Pan, Guanghui Wu

**Affiliations:** School of Chemistry and Chemical Engineering, Qilu Normal University, Jinan 250200, China; zzqchemistry2027@163.com (Z.Z.); pdf5207@126.com (D.P.); wghaerogel2027@163.com (G.W.)

**Keywords:** polymethylsilsesquioxane, polyimide, aerogel, thermal insulation

## Abstract

The inherent brittleness of silica aerogels severely limits their practical application in thermal protection. To address this issue, polymethylsilsesquioxane (PMSQ) wet gels were fabricated via an aqueous sol–gel method, and polyimide (PI) was introduced through in situ polymerization to construct an inorganic–organic continuous framework with PI in situ bridging and interfacial coating, thus successfully preparing the Si/PI composite aerogel. This composite achieves synergistic enhancements in mechanical, thermal insulation, hydrophobic and flame-retardant properties. The flexible PI network effectively alleviates stress concentration, and the compressive modulus of the composite aerogels increased with the PI content, reaching 7.52 MPa for Si/PI-80 and 23.78 MPa for Si/PI-100. The thermal conductivity stays below 0.036 W·m^−1^·K^−1^ with favorable low thermal conduction stability. The Si–CH_3_ groups in PMSQ provide excellent hydrophobicity with a maximum water contact angle of 145.6°. Meanwhile, the char-forming effect of PI at high temperatures counteracts the negative impact of methyl groups on flame retardancy, endowing the aerogel with self-extinguishing behavior and high thermal stability. This work offers a novel strategy for designing high-strength multifunctional silica-based aerogels, which exhibit promising application prospects in aerospace, building energy conservation and high-end thermal protection.

## 1. Introduction

With the growing demand for high-efficiency thermal insulation materials in modern industry, aerogels have become a research hotspot owing to their unique nanoporous structure. As a typical representative, silica aerogels present core advantages including low density, high porosity, and ultra-low thermal conductivity [1,2,3]. These structural merits endow them with exceptional thermal insulation properties, showing great application potential in aerospace, building energy conservation, and thermal protection of high-end equipment [4,5]. However, restricted by the weak interfacial bonding between nanoparticles, silica aerogels generally suffer from severe brittleness [6]. This defect leads to fragmentation, pulverization, and structural collapse under external force, which seriously restricts their practical engineering applications in scenarios with strict mechanical property requirements [6,7]. Therefore, developing silica-based composites with both excellent thermal insulation and mechanical strength has become the key to breaking through their application bottlenecks [8].

At present, the common strategies to improve the mechanical properties of silica aerogels mainly include doping reinforcement [9,10] and silicon-source copolymerization [11,12]. Among them, doping reinforcement is a modification method that introduces fibers [13,14], nanoparticles [15,16], and other components into the aerogel matrix to enhance mechanical and thermal stability through interfacial bonding and structural synergistic effects. For example, Zhao et al. [17] confirmed that the introduction of basalt fibers can effectively improve the mechanical properties of silica aerogels. Yang et al. [16] successfully achieved the synergistic improvement of high-temperature stability, thermal insulation performance, and low density of aerogels by doping hollow SiO_2_ nanoparticles. Nevertheless, poor interfacial compatibility commonly exists between inorganic reinforcing phases and siloxane matrices [18].

Studies have shown that compared with doping reinforcement, silica aerogels prepared by silicon-source copolymerization have denser particle bonding and stronger interfacial interactions [19,20,21,22]. This method constructs a hybrid framework network via co-hydrolysis and polycondensation by regulating the silicon-source structure and copolymerization ratio, which can significantly improve the mechanical properties of materials [22]. For instance, Luo et al. [23] prepared high-temperature-resistant thermal insulation aerogels with both low density and high compressive strength by copolymerizing organoalkoxysilane with polymethyldimethoxysiloxane. Wang et al. [24] modified MTMS-based aerogels with polymethyldimethoxysiloxane as a multifunctional reinforcing agent, endowing the materials with excellent hydrophobicity and thermal stability. However, this strategy significantly deteriorates the flame retardancy of aerogels due to the introduction of flammable organic groups such as methyl groups, greatly limiting their application in high-temperature thermal protection systems [25]. To address the bottleneck of insufficient heat resistance and flame retardancy of traditional polymers, polyimide (PI) demonstrates irreplaceable application potential in high-temperature protection and aerospace fields by virtue of its excellent flame-retardant properties and outstanding thermal insulation performance [26,27].

**Figure 1:**
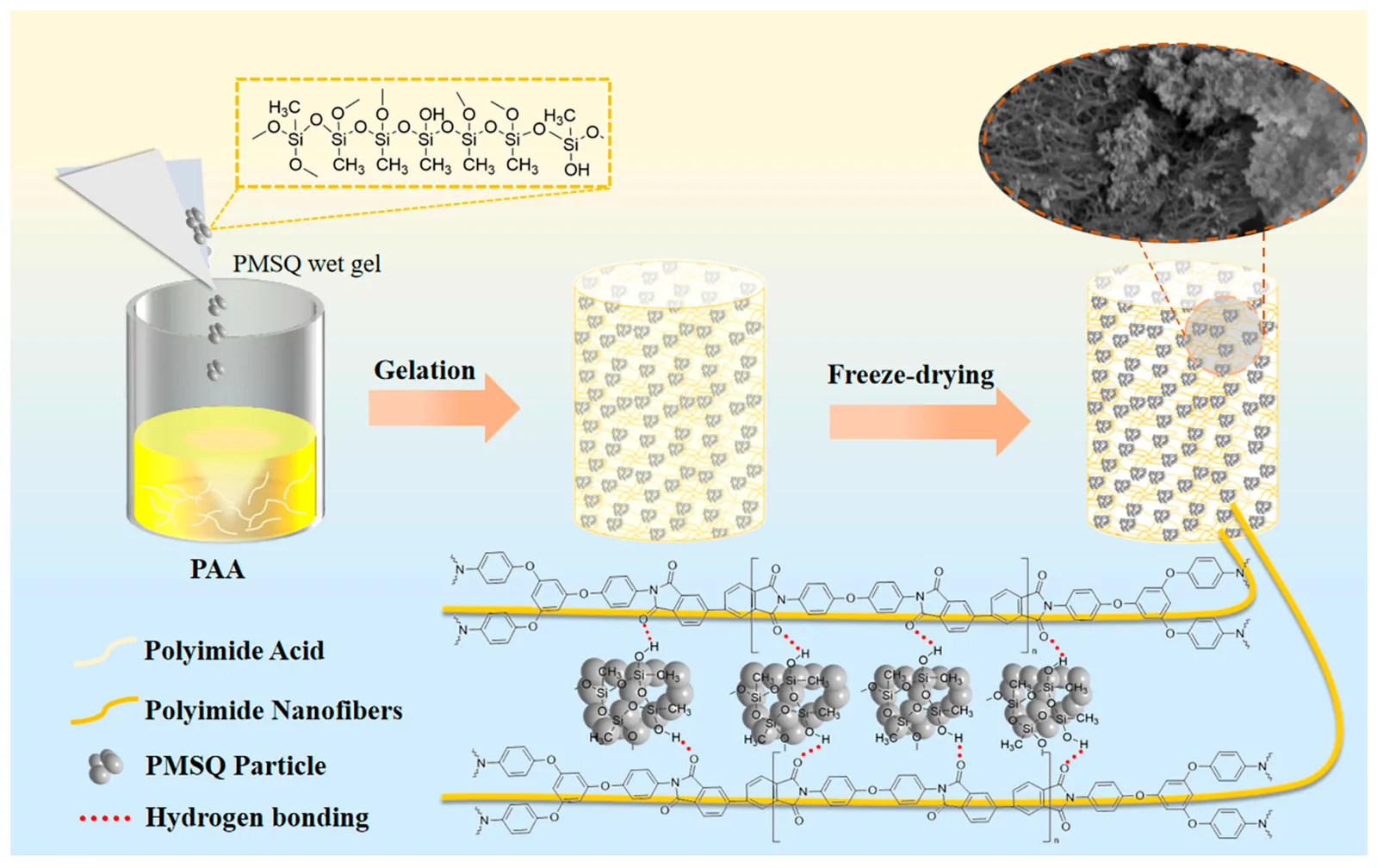
Schematic of the fabrication of Si/PI composite aerogels via in situ polyimide bridging-coating strategy.

Here, PI was selected as a binder to composite with polymethylsilsesquioxane (PMSQ) wet gel powder through in situ imidization. An inorganic–organic integrated composite aerogel with PI nanofiber bridging and coating structures was successfully prepared, as shown in Figure 1. This structure is centered on a stable PI nanofiber-bridged framework and PMSQ particles and features abundant nanoscale pores. While moderately adjusting the compactness to strengthen mechanical properties, it still retains the lightweight characteristic and excellent thermal conductivity of aerogels. The obtained composite aerogel possesses favorable hydrophobicity, outstanding flame retardancy, and reliable mechanical properties simultaneously. In addition, traditional silica aerogels are mostly prepared using organic solvents such as ethanol and toluene as reaction media, which not only increase production costs but also pose operational safety hazards. In this study, only water was used as the reaction medium in the synthesis of PMSQ wet gels, without the need for a large number of volatile organic solvents, making the process simpler, more economical, greener, and safer. The combination of the economical and efficient preparation process and excellent multifunctional properties makes this composite aerogel a promising high-efficiency thermal insulation material.

## 2. Results and Discussion

### 2.1. Microstructure and Component Analysis

PI was used as the interfacial bonding and reinforcing phase to achieve efficient adhesion and compositing of PMSQ wet gel powders through an in situ imidization process. Si/PI composite aerogels with an inorganic–organic continuous integrated framework featuring PI in situ bridging and particle coating were successfully prepared, whose macroscopic morphology is shown in Figure S1. The results indicate that the concentration of polyamic acid (PAA) exerts a significant regulatory effect on the macroscopic morphology and physical properties of the composite aerogels (Table 1). With increasing PAA concentration, the apparent density of the material gradually rises from 112 mg·cm^−3^ to 239 mg·cm^−3^, while maintaining excellent lightweight characteristics overall. This change stems from the increased solid content in the composite system caused by the higher PAA loading, which intensifies shrinkage during drying and thus significantly improves the material’s compactness. When the PAA concentration is as low as 40 mg·cm^−3^, the sample exhibits low mechanical strength, is prone to fragmentation, and shows poor formability. The main reason is that the low-concentration precursor solution has insufficient viscosity, failing to stably disperse and support PMSQ particles. Local agglomeration of particles occurs easily, preventing the formation of a uniformly continuous particle-lapping and PI-bridging structure, which ultimately leads to a remarkable decrease in structural strength and stability. As the PAA concentration is increased to 80 mg·cm^−3^, the Si/PI-80 sample presents a regular and uniform morphology with significantly improved mechanical properties. This is because an appropriate PAA concentration ensures sufficient in situ formation of PI in the interparticle gaps, forming stable interfacial interactions with the surfaces of PMSQ particles and achieving effective particle coating and interparticle bridging. Consequently, a composite porous system with a continuous structure, dense framework, and outstanding mechanical bearing capacity is constructed [28]. Therefore, rational regulation of the PAA concentration enables the synergistic optimization of framework continuity, compactness, and interfacial bonding strength, which is the key to obtaining Si/PI composite aerogels with excellent comprehensive physicochemical properties.

**Figure 2:**
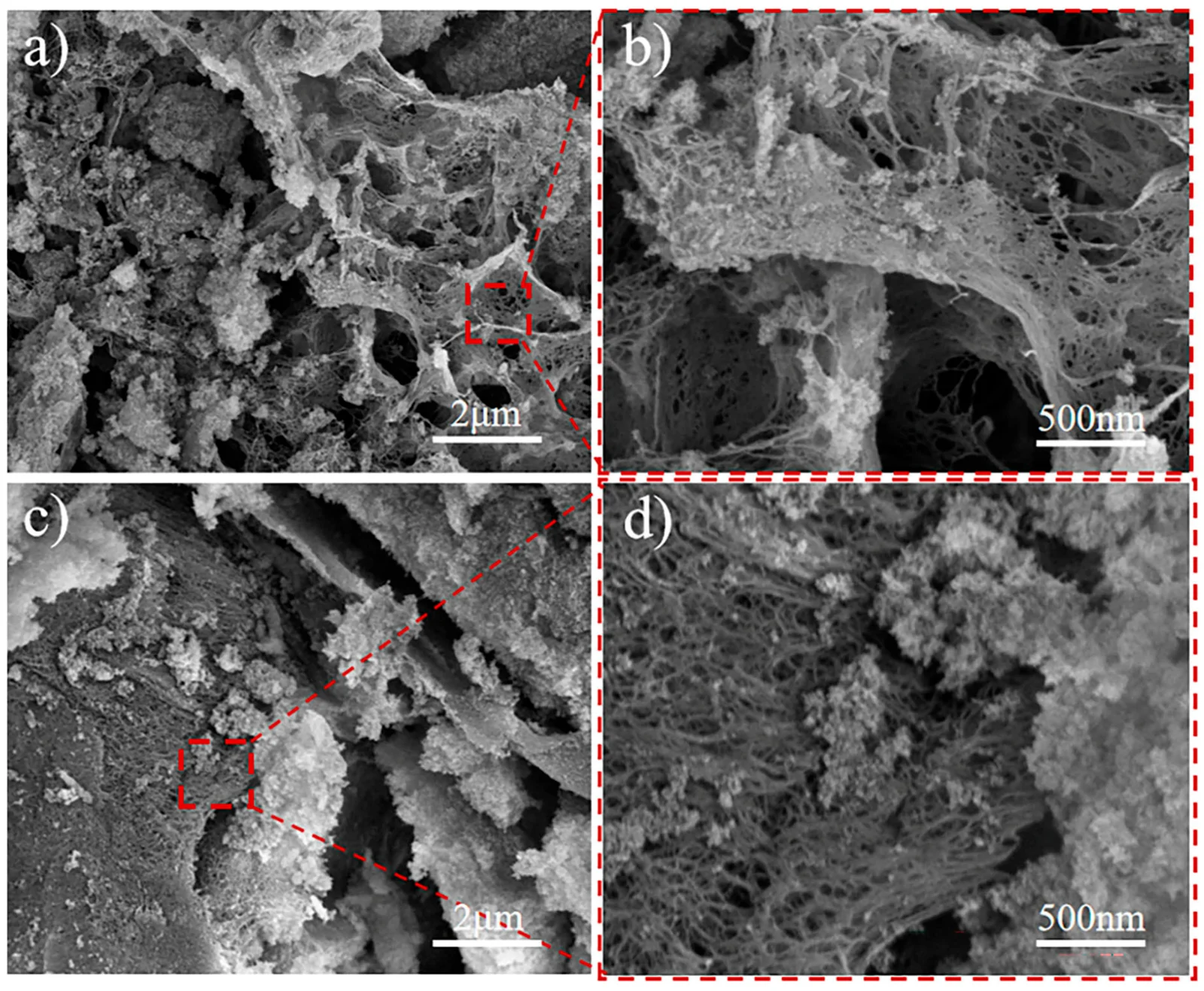
Microstructural evolution of Si/PI composite aerogels at different PAA concentrations. (**a**,**b**) SEM images of Si/PI-40 at scales of 2 μm and 500 nm, showing insufficient PI bridging and weak interfacial bonding. (**c**,**d**) SEM images of Si/PI-80 at scales of 2 μm and 500 nm, demonstrating uniform PI coating, robust interparticle bridging, and a compact continuous porous framework. The red dotted boxes and lines indicate the selected regions for high-magnification observation.

To further reveal the regulation mechanism of PAA concentration on the microstructure of composite aerogels, the micromorphology of Si/PI composite aerogels was characterized, as shown in Figure 2. It was found that the variation in PAA concentration can significantly induce the evolution of the microstructure of composite aerogels (Figure 2 and Figure S2). At a PAA concentration of 40 mg·cm^−3^, only a small amount of PI is generated in the system, which is insufficient to form effective bridging and coating between PMSQ wet gel particles. This results in weak interfacial bonding between PI nanofibers and PMSQ particles, as well as insufficient overall strength of the composite framework. Nevertheless, benefiting from the inherent porous nature of PMSQ, the material still retains a nanoscale porous structure (Figure 2a,b), which is consistent with the macroscopic characteristics of low density but poor mechanical properties at this concentration. When the PAA concentration is increased to 80 mg·cm^−3^, sufficient PI nanofibers are produced via in situ imidization due to the appropriate PAA content. These PI nanofibers can uniformly coat the surface of PMSQ nanoparticles and form continuous bridging structures in the interparticle gaps, significantly improving the compactness and integrity of the composite framework and further enhancing the structural strength (Figure 2c,d). The formation mechanism of this excellent microstructure can be summarized as follows: after PAA is converted into PI nanofibers through in situ imidization, it binds tightly to the surface of PMSQ wet gel particles via intermolecular forces. Through the coating effect and interparticle bridging effect of PI, a continuous three-dimensional porous framework composed of PI nanofibers and PMSQ nanoparticles is self-assembled [29]. Adjusting the PAA concentration can effectively regulate the generation amount and distribution state of PI, strengthen the interfacial interaction between PI and PMSQ phases, and further optimize the microstructure and overall strength of the composite framework, providing structural support for the outstanding mechanical properties of the composite aerogels. Notably, the trifunctional monomer TAB serves as a covalent cross-linker in this system. During PAA polymerization, its three amino groups react with dianhydride to introduce covalent cross-linking points into polyimide molecular chains. The cross-linked network restrains excessive mobility of polymer chains, favors the formation of continuous PI nanofibrous networks, and improves the structural integrity of the resulting composite aerogels.

**Figure 3:**
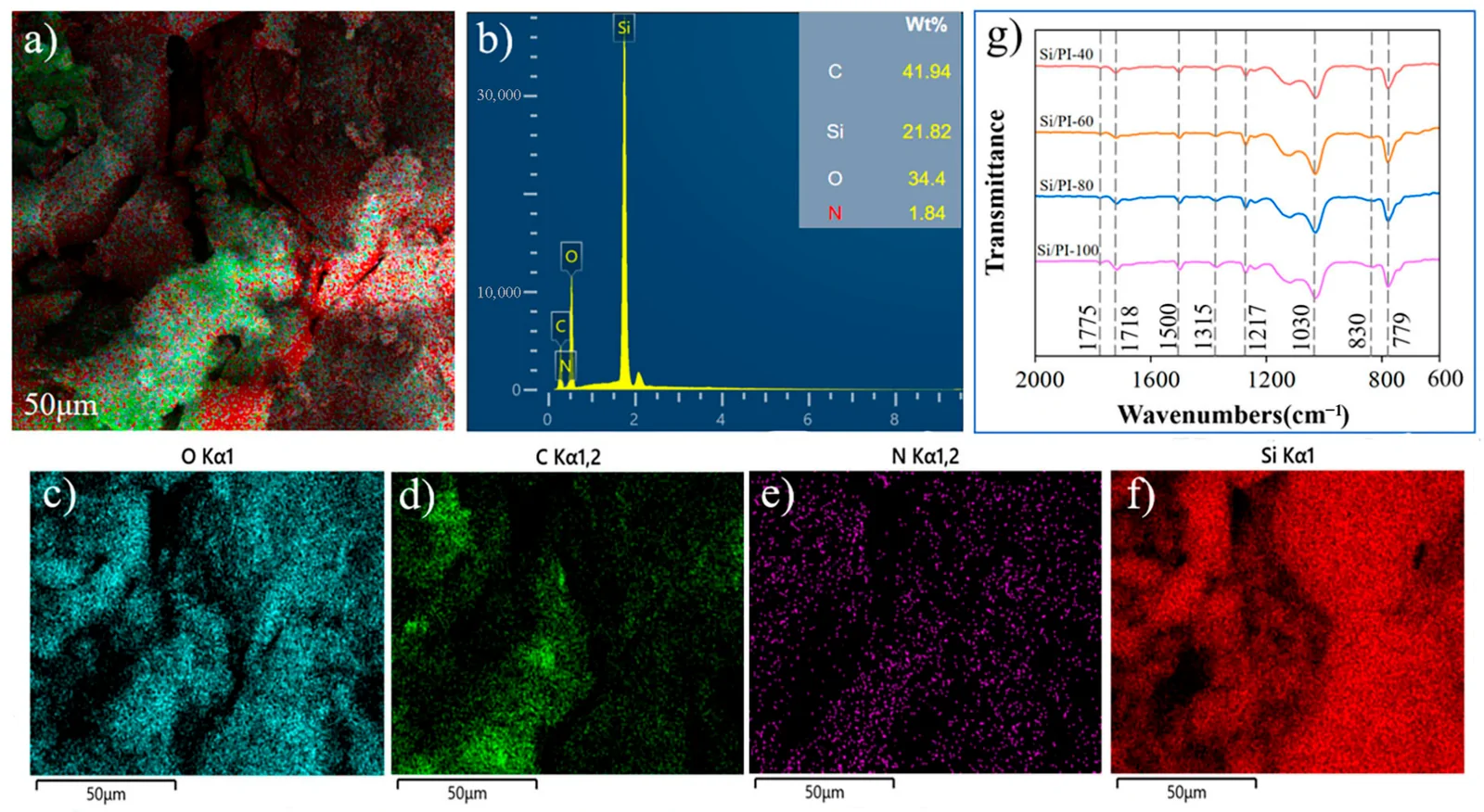
Elemental distribution and chemical composition of the Si/PI-80 composite aerogel. (**a**) SEM morphology. (**b**) Elemental content statistics. (**c**–**f**) EDS mapping of O, C, N, and Si elements, verifying homogeneous dispersion of PMSQ and PI phases without agglomeration. (**g**) FTIR spectra.

To further clarify the elemental composition and spatial distribution characteristics of the composite aerogels, energy-dispersive X-ray spectroscopy (EDS) was systematically used for characterization and analysis to verify the successful introduction of PI and the dispersion uniformity of the two phases. The EDS test results in Figure 3a,b show that four characteristic elements, Si, O, C, and N, could be clearly detected in the Si/PI-80 sample, with corresponding contents of 21.8%, 34.4%, 41.9%, and 1.8%, respectively. Among them, the N element is a characteristic element of the PI molecular chains, and its detection directly confirms that PI has been successfully introduced into the composite aerogel system. Combined with the micro-morphology of the Si/PI-60 sample (Figure 3c,d) and the corresponding elemental mapping results (Figure 3e,f), it can be seen that the Si and O elements are mainly distributed within the PMSQ inorganic particles and are uniformly dispersed, confirming the integrity of the PMSQ skeleton. In contrast, the C and N elements are mainly enriched in the PI nanofiber phase regions and are uniformly distributed without obvious agglomeration. The above elemental distribution characteristics fully confirm that the PMSQ inorganic phase and the PI organic phase achieved uniform dispersion in the composite system, and the two phases are closely bound at the interface. This further confirms that the organic–inorganic integrated skeleton completed sufficient bonding and structural homogenization during the aqueous sol–gel and in situ imidization processes, echoing the previous microstructural characterization results. In addition, to verify the chemical structural integrity and the bonding state of the two phases in the composite aerogels, Fourier transform infrared (FTIR) spectroscopy tests were conducted on the Si/PI composite aerogels, and the results are shown in Figure 3g. In the spectra, the characteristic peaks at 779 cm^−1^ and 1030 cm^−1^ correspond to the bending vibration peak and the asymmetric stretching vibration peak of the Si–O–Si bond, respectively, which are typical fingerprint features of the PMSQ inorganic three-dimensional network skeleton [30], confirming that the PMSQ skeleton was not destroyed during the compounding process. The characteristic peak at 1271 cm^−1^ coincides with the stretching vibration peak of the Si–CH_3_ (silyl methyl) group, and the characteristic peak at 830 cm^−1^ is the typical absorption peak of the –CH_3_ (methyl) group, confirming the successful introduction of methyl groups into the composite system. This further indicates that the composite system has formed a methyl-rich siloxane network structure, which is the core reason for the excellent hydrophobicity of the aerogel [31]. Meanwhile, all characteristic peaks of PI are clearly presented in the spectra: the symmetric and asymmetric stretching vibration characteristic peaks of the C = O bond in the imide ring appear at 1718 cm^−1^ and 1775 cm^−1^, respectively, and the stretching vibration characteristic peaks of the C–N bond and the aromatic ring C = C bond are located at 1375 cm^−1^ and 1500 cm^−1^, respectively. This confirms that the core molecular structure of PI was not destroyed during the preparation process of the composite aerogel and always maintained its complete chemical structural characteristics [32]. Combined with the detection results of the Si–O–Si bond characteristic peaks mentioned above, it is further confirmed that PI formed a tight interfacial bond with the PMSQ matrix through in situ imidization, successfully constructing a structurally stable Si/PI composite system, which provides reliable chemical structural support for the improvement of the material’s comprehensive properties.

To further reveal the regulatory effect of the PAA concentration on the pore architecture of the composite aerogels, nitrogen adsorption–desorption measurements were conducted (Figure S3). All samples exhibit IUPAC Type IV (a) isotherms with H3-type hysteresis loops, indicating a mesoporous structure formed by particle stacking. The BET specific surface areas are 466.43, 503.36, 398.06 and 406.68 m^2^/g for Si/PI-40, Si/PI-60, Si/PI-80 and Si/PI-100, respectively, and the average pore diameters are 18.89, 18.96, 19.53 and 31.15 nm. At a moderate PAA loading, the specific surface area first increases (Si/PI-60), whereas at higher loadings it decreases owing to partial network agglomeration and pore collapse; in contrast, Si/PI-100 exhibits a markedly increased average pore diameter due to abundant inter-particle macropores. These pore-structure results confirm the presence of abundant mesopores inside the material, which suppress gas-phase heat conduction via the Knudsen effect and thus favor a low thermal conductivity.

### 2.2. Mechanical Properties

**Figure 4:**
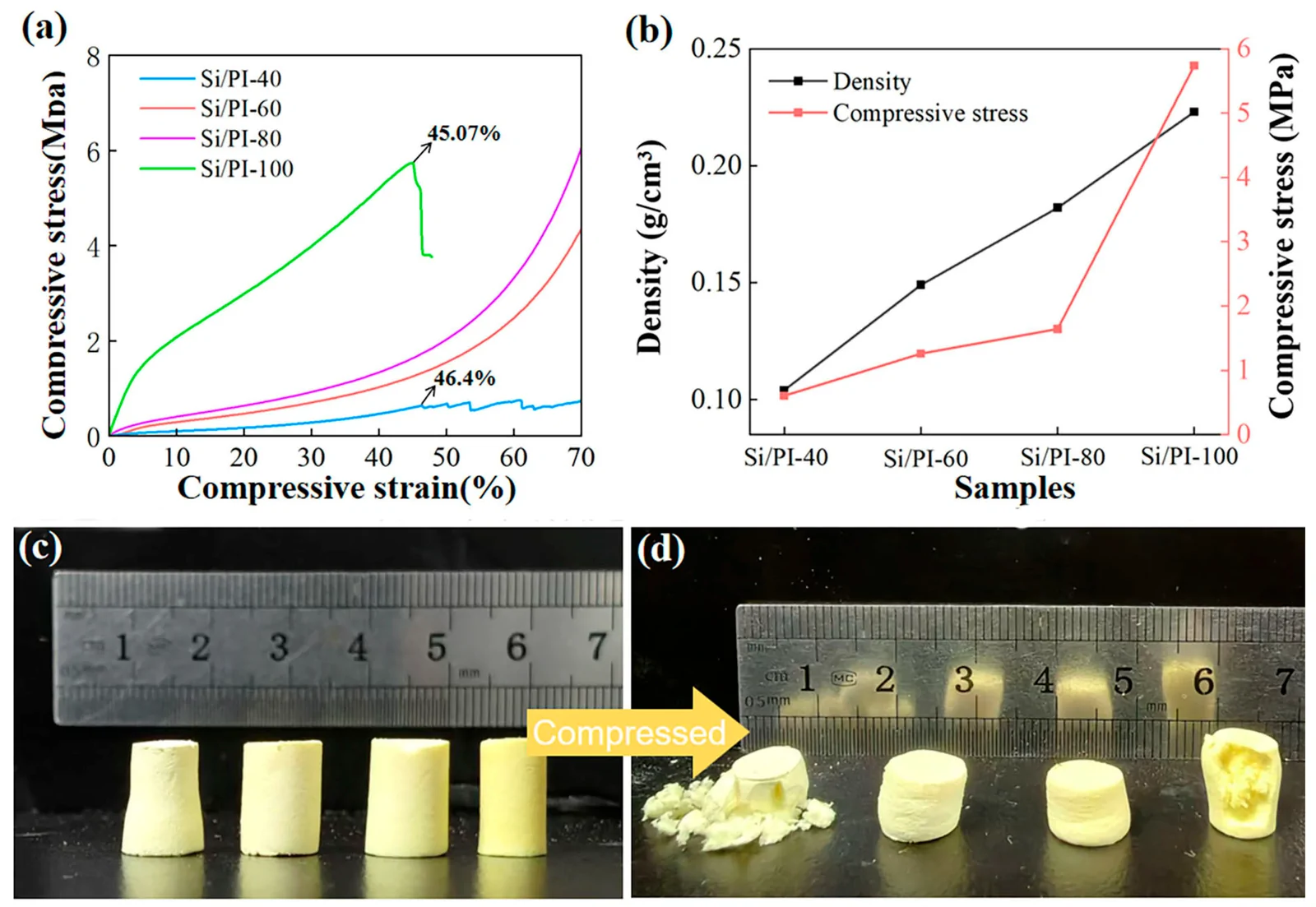
Compressive mechanical behavior and structure–strength relationship of Si/PI composite aerogels. (**a**) Stress–strain curves under uniaxial compression, revealing the brittle–ductile transition regulated by PI content. (**b**) Variations of bulk density and compressive strength at 43% strain, identifying the optimal composition. (**c**) Digital photographs of original samples. (**d**) Morphologies after 70% strain compression, demonstrating structural robustness.

To systematically investigate the compressive mechanical behavior of Si/PI composite aerogels, uniaxial compression tests were conducted on a series of samples using an electronic universal mechanical testing machine. The results demonstrate that the as-prepared composite aerogels exhibit significantly improved mechanical strength and structural stability. As shown in Figure 4a, both Si/PI-60 and Si/PI-80 samples can withstand 70% deformation while maintaining structural integrity. Among them, Si/PI-80 achieves a compressive strength of 6.085 MPa at 70% strain, showing excellent mechanical performance. The mechanical reinforcement mechanism can be attributed to strong hydrogen bonding formed between in situ generated PI molecular chains and hydroxyl groups on the surface of PMSQ particles, enabling PI nanofibers to tightly coat and bridge PMSQ particles. Under external loading, stress is efficiently dispersed, large deformation is accommodated, and stress transfer and dissipation are promoted, thereby significantly enhancing the toughness and load-bearing capacity of the composite [6,33].

The PI content directly determines the integrity and fracture behavior of the composite network. Si/PI-40 exhibits low stress throughout compression and fractures at 46.4% strain, owing to insufficient PI content that fails to form a continuous cross-linked network and results in weak interparticle interfacial bonding. In contrast, Si/PI-100 undergoes brittle fracture at 45.07% strain because excessive PI leads to over-densification of the system, restricted movement of polymer segments, and increased brittleness. Therefore, a moderate PI content is crucial for constructing a stable inorganic–organic composite network and achieving high compression resistance and deformation tolerance. Figure 4b plots the apparent density and compressive modulus of the Si/PI composite aerogels with different PI contents. The compressive modulus was determined by linear fitting of the linear-elastic region (2–4% strain) of the stress–strain curves, providing a strain-independent metric for mechanical stiffness. With increasing PI content, the apparent density increases linearly from 0.112 g·cm^−3^ (Si/PI-40) to 0.239 g·cm^−3^ (Si/PI-100), caused by aggravated framework shrinkage during drying at higher PI loading. Correspondingly, the compressive modulus rises monotonically with PI content, from 1.13 MPa (Si/PI-40) to 23.78 MPa (Si/PI-100). Likewise, the compressive stress at a unified 43% strain increased monotonically with PI content, from 0.24 MPa (Si/PI-40) to 5.01 MPa (Si/PI-100), consistent with the modulus trend. This parallel enhancement in both stiffness and load-bearing stress confirms that the PI network effectively reinforces the aerogel framework while alleviating stress concentration. Notably, Si/PI-100, despite exhibiting a higher failure strain than the brittle compositions, delivers the highest compressive modulus, confirming the most effective reinforcement by the PI network. The macroscopic morphologies of all samples before and after compression are displayed in Figure 4c,d.

These results confirm that tuning PI content to an optimal range can effectively strengthen the framework structure via PI bridging and coating while preserving the lightweight nature of aerogels. This approach markedly alleviates the inherent brittleness of PMSQ aerogels and provides reliable structural support for their applications in thermal protection.

### 2.3. Thermal Insulation Properties

**Figure 5:**
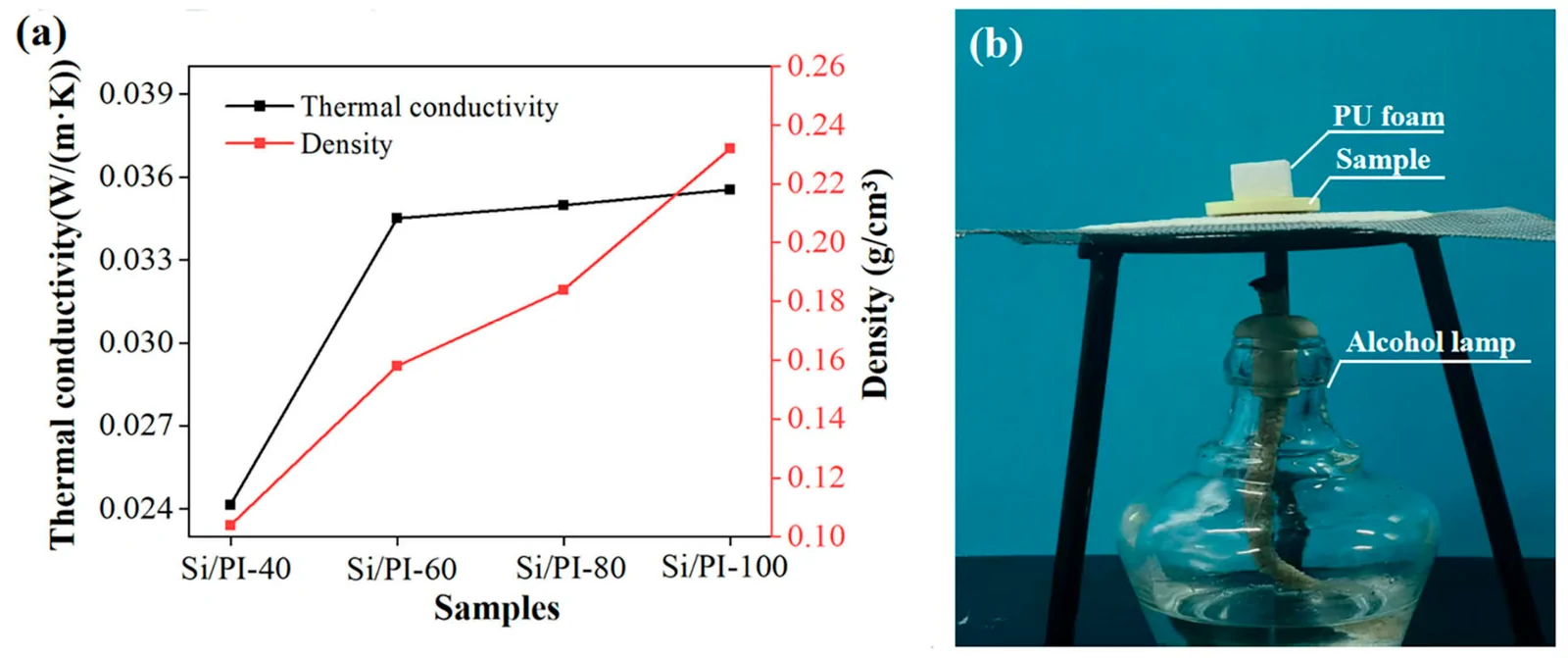
Thermal conductivity and high-temperature thermal insulation behavior of Si/PI composite aerogels. (**a**) Correlation between bulk density and thermal conductivity. (**b**) Schematic diagram of thermal barrier test under alcohol lamp heating.

To investigate the thermal insulation properties of the composite materials, thermal conductivity tests were carried out on a series of samples in this study. As shown in Figure 5a, the thermal conductivity of all samples is below 0.036 W·m^−1^·K^−1^, indicating that the as-prepared composite aerogels possess outstanding thermal insulation performance. This property benefits from their unique hierarchical nanoporous structure: on one hand, the abundant mesopores inside the material are smaller than the mean free path of air molecules, which can significantly suppress the thermal motion and heat transfer of gas molecules; on the other hand, the tortuous and extended heat transfer paths can effectively dissipate heat and further reduce the overall heat conduction efficiency [34,35]. With the increase in PAA concentration, the thermal conductivity of the samples shows a slow upward trend and finally reaches 0.0355 W·m^−1^·K^−1^. This is attributed to the increased compactness of the composite aerogels and the more continuous and dense inorganic–organic framework caused by the higher PI content, which increases the solid-phase heat conduction paths [31]. Notably, the thermal conductivity of the Si/PI-40 sample is as low as 0.0242 W·m^−1^·K^−1^, which is significantly lower than that of the other samples. This abnormally low value may result from test deviation: the sample has weak mechanical strength and is relatively brittle, and its molded size during testing is too small to fully cover the working surface of the instrument probe, leading to a low measured thermal conductivity. In addition, this system greatly enhances the mechanical properties by moderately increasing the material density without causing a significant rise in thermal conductivity, successfully realizing the synergistic optimization of mechanical and thermal insulation properties. Although the introduction of PI improves the compactness of the composite system and moderately reduces the porosity, PI is in situ filled in the gaps between PMSQ particles, constructing a particle composite framework bridged and coated by PI. This framework is continuous and rich in nanopores, which can effectively improve mechanical strength, fully confine air molecules, inhibit gas thermal convection, and maintain the overall low thermal conductivity by virtue of the low thermal conductivity of PI itself [36]. Therefore, the as-prepared Si/PI composite aerogels still maintain excellent low thermal conductivity stability even with increased density, achieving a balance between mechanics and thermal insulation, and possess prominent application value in the field of high-performance thermal protection.

**Figure 6:**
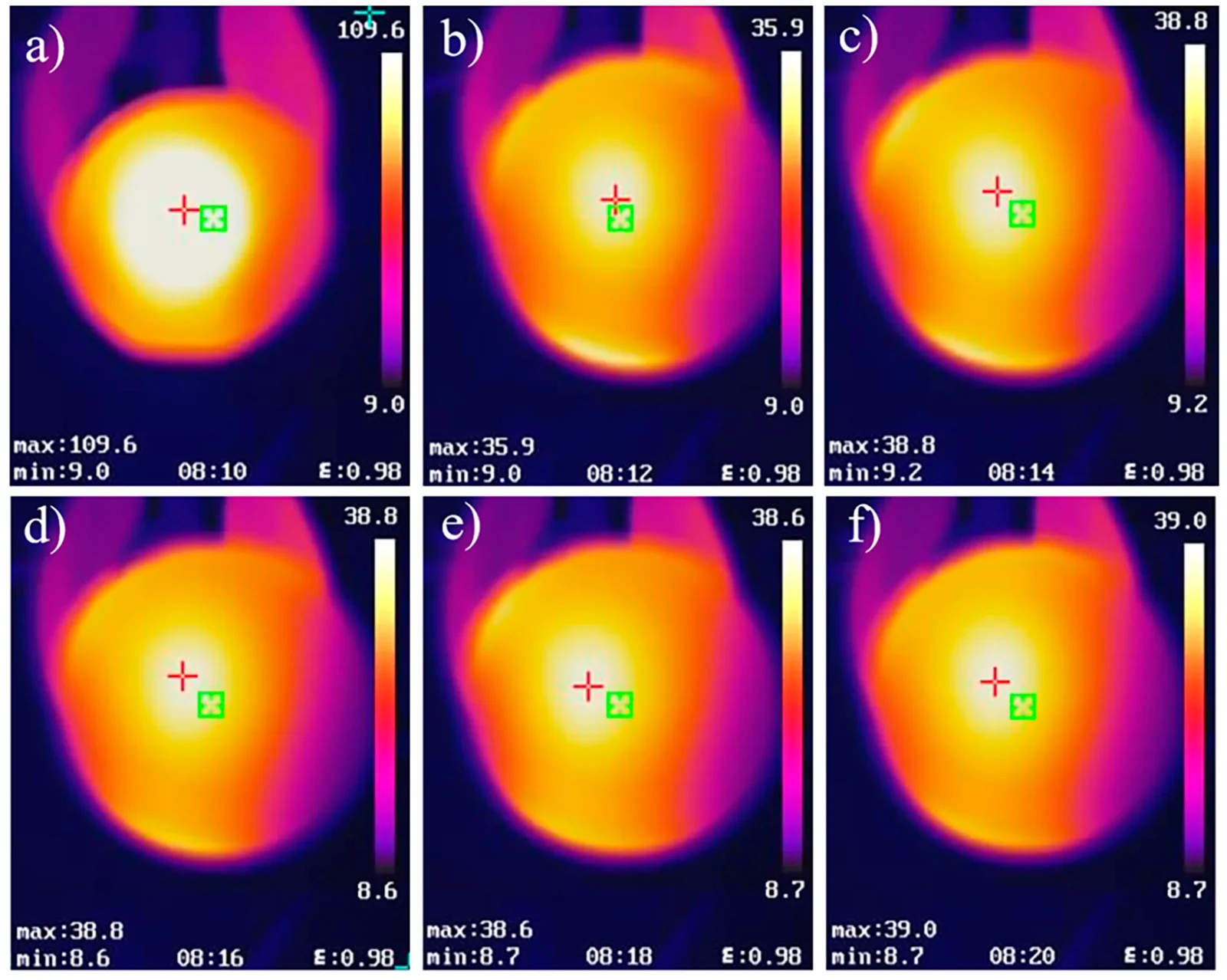
Thermal infrared temperature distribution of Si/PI-60 aerogel under continuous heating. (**a**–**f**) Infrared thermal images recorded at 0, 2, 4, 6, 8 and 10 min, respectively. The red crosshairs denote the maximum temperature spot; the green box marks the field-of-view center.

The thermal insulation performance of the Si/PI composite aerogels was further investigated using a thermal infrared camera. The sample was placed directly above and in contact with a point heat source maintained at 110 ± 1 °C. The upper surface of the sample was observed with a thermal infrared imager, and the surface temperature was recorded every 2 min to obtain thermal infrared data (Figure 6). After 10 min, the upper surface temperature of Si/PI-60 was only 38.3 °C, indicating that the composite aerogel effectively blocks heat transfer from the high-temperature heat source. This improvement is not attributed to an increase in the porosity of the PMSQ aerogel framework induced by PI but to the in situ filling of PI in the interparticle gaps of PMSQ, forming a continuous and nanoporous dense framework that strongly confines air molecules and suppresses gas-phase heat conduction, thus maintaining efficient thermal barrier capability. In addition, thermogravimetric analysis (TGA) was performed to evaluate the high-temperature thermal stability of the composite aerogels. As shown in Figure S4, the material exhibits a typical two-step thermal decomposition process. Two major thermal degradation peaks appear during heating: the first peak at 598.5 °C corresponds mainly to the high-temperature oxidative decomposition of the Si–CH_3_ organic side groups in the PMSQ network; the second peak at 704.6 °C is attributed to the thermal cleavage of the PI main chain and the decomposition of the framework structure. The TG curve reveals that the initial decomposition temperature of the composite aerogel exceeds 550 °C, demonstrating favorable thermal stability. This is mainly ascribed to the synergistic stabilizing effect of the rigid aromatic structure of PI and the siloxane inorganic framework, which significantly enhances the high-temperature thermal stability of the material [37]. To further verify the high-temperature stability and thermal insulation performance, as illustrated in Figure 5b, expanded polystyrene (EPS) foam with a volume of 1 cm^3^ was placed on top of a 4-mm-thick composite aerogel and heated continuously from below with an alcohol lamp. The results show that under the efficient thermal barrier of the composite aerogel, the EPS foam began to melt after approximately 1 min and retained a partial structure even after 3 min of heating, while the composite aerogel itself remained intact (Video S1). In contrast, the EPS foam melted almost completely within only 18 s under direct heating (Video S2). This further confirms the superior thermal insulation performance and high-temperature stability of the Si/PI composite aerogels. Based on the above results, the composite aerogel shows promising application prospects in the field of thermal protection.

### 2.4. Flame Retardancy

**Figure 7:**
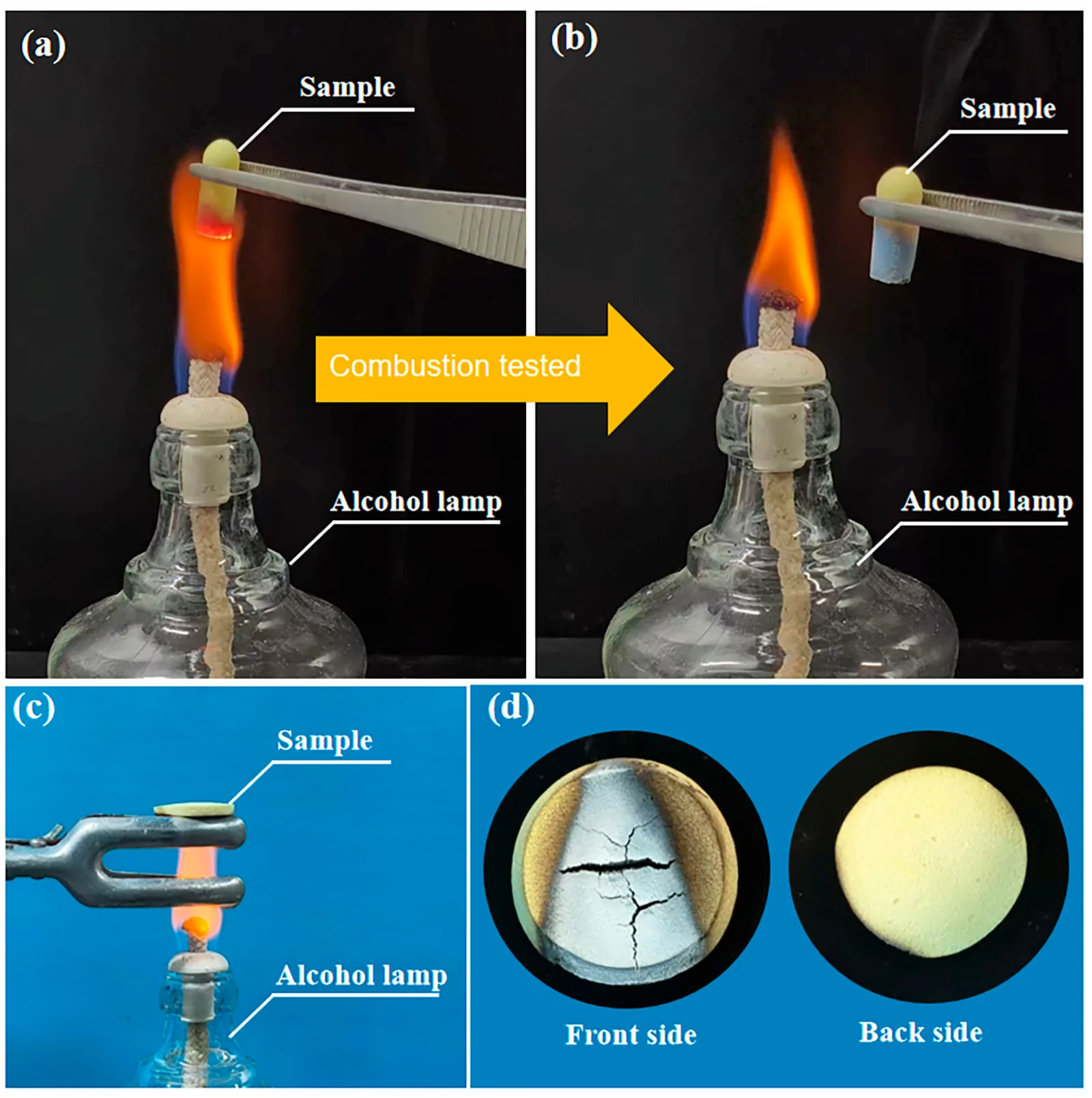
Flame-retardant behavior and ablation resistance of Si/PI composite aerogels. (**a**) Schematic of open-flame combustion test. (**b**) Digital photographs after combustion, highlighting self-extinguishing behavior. (**c**) Schematic of long-duration flame ablation. (**d**) Front/back morphologies after ablation, demonstrating intact structure, negligible carbonization, and excellent flame-shielding capability.

To systematically evaluate the flame retardancy and high-temperature thermal stability of Si/PI composite aerogels, comparative tests on the combustion behavior of the materials were conducted in this study. The composite aerogels and commercial expanded polystyrene (EPS) foam were respectively exposed to the outer flame of an alcohol lamp for combustion testing (Figure 7a). The results show that EPS foam burns violently, melts and collapses rapidly upon exposure to open flame, whereas the as-prepared Si/PI composite aerogels exhibit outstanding flame retardancy and ablation resistance. The Si/PI-80 sample can self-extinguish immediately after removal from the open flame (Figure 7b, Video S4). Although the Si–CH_3_ methyl groups in the PMSQ framework are prone to thermo-oxidative degradation at high temperatures, which may adversely affect the flame retardancy of the material, the introduction of the PI phase effectively offsets this disadvantage. The PI molecular chains are rich in aromatic rings and rigid imide structures, which can rapidly form a continuous and dense protective layer upon heating, effectively isolating oxygen and heat transfer and inhibiting the degradation of methyl groups and the release of flammable small molecules at the source [38,39]. On this basis, the unique three-dimensional nanoporous network of the material further endows it with excellent high-temperature thermal barrier capability: the continuous inorganic–organic composite framework resists thermal shock and ensures structural integrity during combustion; the abundant internal nanopores greatly weaken solid-phase heat conduction and gas thermal convection, significantly delaying heat transfer to the interior and the back-fire surface. After continuous ablation by the outer flame of an alcohol lamp for 5 min, only slight carbonization occurs on the front side of the sample, while the back side retains its original color and morphology (Figure 7c,d, Video S5), fully confirming its exceptional flame retardant stability and synergistic thermal insulation performance. Therefore, the Si/PI composite aerogel effectively suppresses the negative effects on flame retardancy caused by methyl groups in PMSQ through the efficient charring effect of the rigid PI framework, ultimately achieving superior self-extinguishing behavior upon flame removal and high-temperature ablation resistance. It possesses significant application potential in extreme high-temperature thermal protection scenarios.

### 2.5. Hydrophobicity

**Figure 8:**
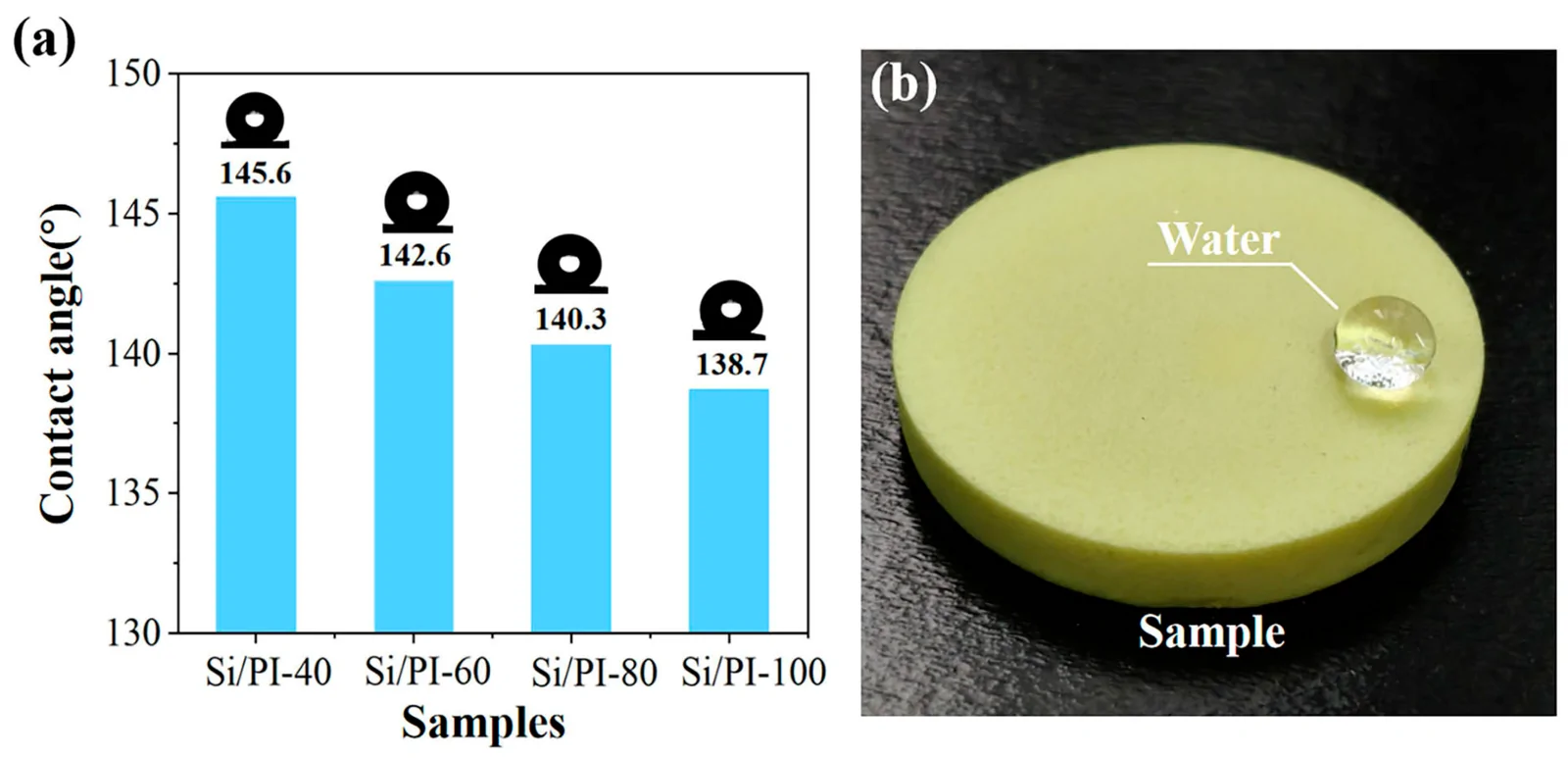
Hydrophobic characteristics and surface wettability of Si/PI composite aerogels. (**a**) Histogram of water contact angles for various samples, showing superior hydrophobicity maintained over a wide PI content range. (**b**) Optical photograph of a water droplet on Si/PI-80 surface, presenting spherical morphology and low surface energy feature.

The hydrophobic properties of the Si/PI composite aerogels were systematically characterized using water contact angle (WCA) measurements in this study. The results indicate that all samples exhibit favorable hydrophobicity, with water contact angles above 130° (Figure 8a). As the PI content in the system increases, the water contact angle of the material shows a slow downward trend, which is mainly related to the inherent polarity and hydrophilicity of the PI molecular chains. Notably, even the Si/PI-100 sample with the highest PI loading retains a water contact angle of 138.7°, maintaining excellent hydrophobic performance. To further verify the hydrophobicity, water droplets were placed on the surface of the Si/PI-80 sample; the droplets remained nearly spherical without spreading or wetting (Figure 8b, Video S6), directly reflecting the low surface energy characteristic of the material surface. The outstanding hydrophobicity mainly originates from the abundant nonpolar Si–CH_3_ methyl groups in the PMSQ framework, which significantly reduce the surface energy and endow the composite aerogels with stable hydrophobic behavior. Although a higher PI content slightly weakens hydrophobicity, the material still maintains excellent hydrophobic performance within the regulation range of this study. It can effectively resist moisture erosion and broaden its application scope in high-humidity environments.

### 2.6. Environmental Durability Tests

To evaluate the practical durability of the Si/PI composite aerogels under service-related conditions, thermal-insulation, humidity-aging and high-temperature treatments were performed, and the results are presented in Figure S5. First, the thermal-insulation performance was assessed by placing the pristine Si/PI-80 specimen in direct contact with a point heat source at 110 ± 1 °C. After heating for 10 min, the upper-surface temperature of Si/PI-80 reached only 40.7 °C (Figure S5a,b), demonstrating its excellent heat-insulating capacity. Subsequently, humidity-exposure aging was carried out. After being stored in a sealed saturated-vapor atmosphere at room temperature for 120 h, the monolithic Si/PI-80 retained its structural integrity and favorable hydrophobicity, with water droplets maintaining a spherical shape on the sample surface. Thermal infrared imaging revealed only minor fluctuations in thermal-insulation performance without obvious degradation (Figure S5c,d). The humidity-treated sample was then placed in a high-temperature oven and thermally treated at 250 °C for 48 h. No obvious structural change was observed, and thermal infrared imaging showed that its thermal-insulating performance exhibited no significant difference compared with the untreated sample (Figure S5e,f). This observation is consistent with the TGA results (Figure S4), which confirm the good thermal stability of the material below 300 °C. In summary, the material exhibits good short-term resistance to high-temperature and humid conditions, indicating its feasibility for practical thermal-insulation applications.

Moreover, unlike the impregnation, co-gelation and physical powder-blending strategies reported in the recent literature (Table S1), in this work the PI precursor served as the liquid phase and the preformed PMSQ as the solid phase, and the two components were combined through a one-step in situ gelation route. The in situ generated polyimide nanofibers wrapped and bridged the silica particles, constructing a compact organic–inorganic interface. This one-step approach enables precise control of the PI content via the feeding ratio, circumvents the penetration-depth limitation inherent to thick monoliths, and thereby affords a well-balanced aerogel material that combines large-strain mechanical strength and toughness, hydrophobicity, thermal insulation, and resistance to direct flame ablation. Meanwhile, compared with the PMSQ aerogels reported by Hayase et al. [40] and the guar gum–cellulose foams reported by Ergün et al. [41], the Si/PI-80 aerogel prepared in this work exhibits superior mechanical performance. Benefiting from the intrinsic flame-retardant nature of polyimide, the Si/PI-80 aerogel also possesses reliable ablation resistance, endowing it with outstanding comprehensive application prospects in the field of thermal insulation.

## 3. Conclusions

In summary, PMSQ wet gels were prepared via an aqueous sol–gel method in this work, and PI was introduced into the silicon-based framework through in situ imidization. An inorganic–organic integrated continuous framework with PI in situ bridging and particle coating was successfully constructed, yielding Si/PI composite aerogels with excellent comprehensive performance. PI nanofibers can effectively bond and coat PMSQ particles to form a strong and interlocked network, fundamentally overcoming the brittleness defect of conventional silica aerogels and realizing outstanding mechanical properties under high strain. With increasing PI content, the compactness of the material is moderately improved. Benefiting from the efficient confinement effect of the nanoporous structure on gas heat transfer, the composite aerogels still maintain stable low thermal conductivity, achieving a synergistic balance between mechanical performance and thermal insulation properties. The Si–CH_3_ groups in the PMSQ framework impart excellent hydrophobicity to the material. PI rapidly forms a dense char layer at high temperatures, which effectively counteracts the adverse effects caused by thermo-oxidative degradation of methyl groups, endowing the material with reliable flame retardancy and thermal stability. The results demonstrate that the as-prepared Si/PI composite aerogels integrate multifunctional properties including light weight, high strength, low thermal conductivity, hydrophobicity, and flame retardancy. The preparation process is simple, green, and economical, showing significant engineering application value in the field of high-performance thermal protection.

## 4. Materials and Methods

### 4.1. Materials

Reagents including methyltrimethoxysilane (MTMS, ≥98%), deionized water, ethanol (≥99%), N-methylpyrrolidone (NMP, 98%), 3,3′,4,4′-biphenyltetracarboxylic dianhydride (BPDA, 97%) and 4,4′-oxydianiline (ODA, 98%) were provided by Shanghai Macklin Biochemical Technology Co., Ltd., Shanghai, China. Pyridine (AR grade), acetic acid (AR grade) and tert-butanol (AR grade) were purchased from Tianjin Fuyu Fine Chemical Co., Ltd., Tianjin, China. Dehydrating agent acetic anhydride (AR grade) and sodium hydroxide (NaOH, AR grade) were purchased from Sinopharm Chemical Reagent Co., Ltd. (Shanghai, China). 1,3,5-tris(4-aminophenoxy)benzene (TAB, ≥98%) was purchased from Shanghai Dibo Biotechnology Co., Ltd., Shanghai, China.

### 4.2. Methods

(1) Preparation of PMSQ Wet Gels: PMSQ wet gels were prepared via the sol–gel method using water as the sole solvent. An amount of 12 mL of methyltrimethoxysilane, 15 mL of water and 0.075 mL of acetic acid were mixed at room temperature and magnetically stirred for 30 min. Subsequently, 1 mol/L sodium hydroxide solution was added dropwise under stirring to adjust the pH of the system to 9, and gelation occurred after standing. The gel samples were aged at 70 °C for 8 h and crushed through a 50-mesh standard sieve; the obtained wet gel powders were washed with water three times, then immersed in N-methylpyrrolidone (NMP) for three solvent exchanges, followed by suction filtration for later use.

(2) Preparation of Si/PI Composite Aerogels: 31 mL of NMP solvent was placed in a beaker, and 2 g of ODA was added under magnetic stirring, after ODA was completely dissolved, to form a uniform and transparent clear solution; 3.12 g of BPDA was slowly added to the above solution in batches, and magnetic stirring was continued for a period of time. After the reaction system returned to uniform and transparent again, 0.08 g of TAB, a trifunctional covalent cross-linker, was slowly added under stirring, mixed well and stood at room temperature for 12 h to prepare a stock polyamic acid (PAA) solution. Then, 7.48 mL of acetic anhydride and 6.44 mL of pyridine were added as chemical imidization reagents. The mixture was gradient-diluted rapidly with NMP to obtain precursor solutions with concentrations of 100 mg·cm^−3^, 80 mg·cm^−3^, 60 mg·cm^−3^ and 40 mg·cm^−3^, respectively. Equal masses of each diluted precursor solution and PMSQ wet-gel powder were weighed, mixed thoroughly, and poured into prefabricated molds. The whole operation was completed quickly to suppress excessive imidization in the liquid phase, and the mixture was kept at room temperature for molding. After gel formation upon casting, most of the chemical imidization took place in situ in the particle gaps to produce PI for bridging and coating the PMSQ particles. Solvent replacement was carried out with tert-butanol/water (volume ratio 1:2), and composite aerogels were finally formed after freeze-drying. The samples were named Si/PI-*n* (where *n* = 40, 60, 80, 100, representing the concentration value of the corresponding PAA solution), and the unit mg·cm^−3^ denotes the mass of polyamic acid (PAA) solute per volume of liquid NMP-based PAA precursor solution.

### 4.3. Characterization

The morphology and structure of the samples were characterized using Field Emission Scanning Electron Microscopy (SEM; model: TESCAN MIRA LMS, TESCAN, Brno, Czech Republic). This SEM was equipped with an XFlash FlatQUAD Energy Dispersive X-ray Spectrometer (Bruker, Berlin, Germany). The chemical structure of the samples was characterized using a Fourier transform infrared (FTIR) spectrometer (FTIR ALPHA, Bruker, Berlin, Germany) at a resolution of 4 cm^−1^. The mechanical compressive properties of the samples were measured using a WDW-D1000N Universal Testing Machine (Jinan Xinguang Testing Machine Manufacturing Co., Ltd., Jinan, China). The compression specimens were cylindrical, with a nominal diameter of approximately 7 mm, a nominal height of about 10 mm, and an aspect ratio of ca. 1.4–1.5. The thermal decomposition behavior of the samples in an air atmosphere was studied using a TGA5500 Thermogravimetric Analyzer (manufactured in New Castle, DE, USA). The samples were placed in ceramic crucibles and heated from 20 °C to 800 °C at a heating rate of 10 °C/min. The thermal conductivity of the samples was evaluated using a TRI-4C Thermal Conductivity Analyzer (C-Therm Technologies Ltd., Fredericton, NB, Canada) via the Modified Transient Plane Source (MTPS) method. The thermal decomposition behavior of the samples in a nitrogen atmosphere was investigated using a Hitachi STA200 thermogravimetric analyzer (manufactured in Japan). The samples were placed in a ceramic crucible and heated from 30 °C to 800 °C at a heating rate of 10 °C·min^−1^. The water contact angle of the samples was measured using an OCA 50 contact angle measurement system (DataPhysics, Filderstadt, Germany). Infrared imaging was performed using an H10 Thermal Infrared Camera (Hangzhou Hikmicro Sensing Technology Co., Ltd., Hangzhou, China) aimed at the sample to capture images. The specific surface area and pore-size distribution were measured with an ASAP 2460 analyzer (Micromeritics, Norcross, GA, USA) using nitrogen adsorption–desorption and analyzed by the BET and BJH methods.

## Figures and Tables

**Table 1:** Physical properties of composite aerogels.

Sample Name	Density (mg·cm^−3^)	Thermal Conductivity (W·m^−1^·K^−1^)	Water Contact Angle (°)	Compressive Modulus (MPa)	Toughness Within 43% Strain
Si/PI-40	112 ± 10	0.0242 ± 0.0005	145.6	1.13 ± 0.19	0.066 ± 0.025
Si/PI-60	162 ± 11	0.0347 ± 0.0008	142.6	4.18 ± 0.75	0.257 ± 0.024
Si/PI-80	182 ± 4	0.03497 ± 0.00006	140.3	7.52 ± 2.92	0.391 ± 0.076
Si/PI-100	239 ± 15	0.0355 ± 0.0002	138.7	23.78 ± 2.75	1.127 ± 0.198

Note: The compressive modulus was calculated by linear fitting over the 2–4% linear-elastic strain range of the stress–strain curves. The toughness corresponds to the integrated area under the stress–strain curves from 0 to 43% strain, representing the energy-absorption capacity of the materials.

## Data Availability

The data presented in this study are available on request from the corresponding author.

## Supplementary Materials

The following supporting information can be downloaded at: https://www.mdpi.com/article/10.3390/gels12100898/s1, Video S1: Video of Heating Process for Aerogel-insulated EPS Foam; Video S2: Video of Direct Heating on EPS Foam; Video S3: Video of the Combustion Process of EPS Foam; Video S4: Video of the Combustion Process of Si/PI-80 Aerogel; Video S5: The Burn-through Resistance Test of Sample Si/PI-80 Aerogel. This video has been sped up to 20X; Video S6: Video of the Demonstration of Si/PI-80 Aerogel’s Hydrophobic Properties; Figure S1: Macroscopic Morphology of Samples (from left to right: Si/PI-40, Si/PI-60, Si/PI-80, Si/PI-100); Figure S2: SEM image of samples (a) Si/PI-40 (b) Si/PI-60 (c) Si/PI-80 (d) Si/PI-100; Figure S3: N_2_ adsorption–desorption isotherms and BJH pore-size distribution of Si/PI composite aerogels; Figure S4: TG curves of samples Si/PI-80 (measured under ambient air conditions); Figure S5: Environmental-stability evaluation of the Si/PI-80 composite aerogel; Table S1: Comparison of physicochemical properties of the Si/PI composite aerogel with previously reported polyimide-based aerogels.

## References

[1] Pan Q., Liu Q., Shi Y., Yang D., Lan Y., Wang T. (2025). A facile strategy for freeze-drying preparation of silica aerogel from sodium silicate. Ceram. Int..

[2] Wang J., Petit D., Ren S. (2020). Transparent thermal insulation silica aerogels. Nanoscale Adv..

[3] Xie T., He Y.-L., Hu Z.-J. (2013). Theoretical study on thermal conductivities of silica aerogel composite insulating material. Int. J. Heat Mass Transf..

[4] Lamy-Mendes A., Pontinha A.D.R., Alves P., Santos P., Durães L. (2021). Progress in silica aerogel-containing materials for buildings’ thermal insulation. Constr. Build. Mater..

[5] Wang Y., Chen Z., Yang L., Xu K., Ai S., Zhang J., He L., Xu F., Shen X., Li M. (2026). Lightweight, strong and insulation melamine carbon foam supported SiO_2_ aerogel for aerospace thermal protection systems. Chem. Eng. J..

[6] Chen J., Pan D. (2025). Robust and hydrophobic silica/polyimide aerogel with pomegranate-like structure for thermal insulation and flame retardancy up to 1300 °C. Molecules.

[7] Ni H., Wang D., Jia R., Liu Y., Wu D., Chang S., Li X., Shi J. (2025). Fast preparation of aluminum silicate fiber reinforced silica aerogel composites with the ultra-low thermal conductivity and high temperature resistance by combustion technology. J. Alloys Compd..

[8] Wang Z., Mo J., Tang L., Shi F., Chen L., Li L., Chen M., Kong Q., Zhou R., Jiang J. (2025). Preparation and thermal-mechanical properties of physical and chemical dual-reinforced SiO_2_ aerogels. J. Non-Cryst. Solids.

[9] Zhang X., Zhang T., Yi Z., Yan L., Liu S., Yao X., Guo A., Liu J., Hou F. (2020). Multiscale mullite fiber/whisker reinforced silica aerogel nanocomposites with enhanced compressive strength and thermal insulation performance. Ceram. Int..

[10] Shang L., Lyu Y., Han W. (2019). Microstructure and thermal insulation property of silica composite aerogel. Materials.

[11] Sun Z., Zhao Z., Kong Y., Ren J., Jiang X., Shen X. (2022). Auto-continuous synthesis of robust and hydrophobic silica aerogel microspheres from low-cost aqueous sodium silicate for fast dynamic organics removal. Gels.

[12] Wang Q., Yu H., Zhang Z., Zhao Y., Wang H. (2020). One-pot synthesis of polymer-reinforced silica aerogels from high internal phase emulsion templates. J. Colloid Interface Sci..

[13] Ghica M.E., Mandinga J.G.S., Linhares T., Almeida C.M.R., Durães L. (2023). Improvement of the mechanical properties of silica aerogels for thermal insulation applications through a combination of aramid nanofibres and microfibres. Gels.

[14] Sharma J., Sheikh J., Behera B.K. (2023). Aerogel composites and blankets with embedded fibrous material by ambient drying: Reviewing their production, characteristics, and potential applications. Dry. Technol..

[15] Guo L., Du M., Li J., Li W., Yang M., Xin G. (2025). Tensile resistance and fracture mechanisms of silica aerogels reinforced by nanotube–graphene hybrid networks. Gels.

[16] Yang M., Si Q., Tang G., Sheng Q., Guo L., Yang R., Li N., Zhang H., Gao Q., Peng F. (2025). High-temperature resistance, lightweight, and thermally insulating silica aerogel via doping hollow silica nanoparticles. ACS Appl. Nano Mater..

[17] Xue Z., Liu Z., Ding Y. (2024). Preparation and physical properties of basalt fiber-reinforced silica aerogels. J. Mater. Sci..

[18] Yang X., Sun Y., Shi D., Liu J. (2011). Experimental investigation on mechanical properties of a fiber-reinforced silica aerogel composite. Mater. Sci. Eng. A.

[19] Zhang S., Wang Z., Ji H., Wang J., Lu K., Xu G., Xiao Y., Ding F. (2023). Mechanically robust nanoporous polyimide/silica aerogels for thermal superinsulation of aircraft. ACS Appl. Nano Mater..

[20] Xu L., Zhu W., Chen Z., Su D. (2023). Double-network MK resin-modified silica aerogels for high-temperature thermal insulation. ACS Appl. Mater. Interfaces.

[21] Gong D., Qu M., Wang X., Ai X., Tang P., Zhao W., Wang X., Bin Y. (2025). Preparation of epoxy-enhanced silica aerogels with thermal insulation and hydrophobicity by ambient pressure drying. ACS Appl. Polym. Mater..

[22] Wang Z., Chen L., Kong Q., Li L., Chen M., Jiang J., Li W., Shi F., Xu Z. (2023). The synthesis and polymer-reinforced mechanical properties of SiO_2_ aerogels: A review. Molecules.

[23] Luo Y., Yan A., Tian H., Niu B., Zhang Y., Wang H., Long D. (2024). A robust silicone aerogel via copolymerization of a difunctional organoalkoxysilane and polymethylmethoxysiloxane for high-temperature thermal insulation. J. Mater. Chem. A.

[24] Wang L., Feng J., Jiang Y., Li L., Feng J. (2019). Elastic methyltrimethoxysilane based silica aerogels reinforced with polyvinylmethyldimethoxysilane. RSC Adv..

[25] Wu X., Shen K., Hu M., Zhou F., Chen Z., Liu Q., Li Z., Li Z. (2024). Experimental investigation of the combustion characteristics and thermal hazards of methylsilyl-modified silica aerogels. Gels.

[26] Mir T.A., Tyagi S., Rathour R., Das A., Ali S.W. (2026). Advancements in the development of polyimide-based aerogels for extreme thermal environments: A critical review. ACS Appl. Mater. Interfaces.

[27] Sun J., Tian X., Zhang D., Zhang R. (2024). Temperature-irrelevant superhydrophobic polyimide–SiO_2_ aerogels with a confined shrinkage strategy for high-temperature thermal protection (>400 °C). Compos. Part A Appl. Sci. Manuf..

[28] Yan P., Zhou B., Du A. (2014). Synthesis of polyimide cross-linked silica aerogels with good acoustic performance. RSC Adv..

[29] Lin Y., Hu S., Wu G. (2019). Structure, dynamics, and mechanical properties of polyimide-grafted silica nanocomposites. J. Phys. Chem. C.

[30] Zhang Y., Shen Q., Li X., Xie H., Nie C. (2020). Facile synthesis of ternary flexible silica aerogels with coarsened skeleton for oil–water separation. RSC Adv..

[31] Wang L., Lian W., Yin B., Liu X., Tang S. (2024). Silica nanowires-reinforced silica aerogels with outstanding thermal insulation, thermal stability and mechanical properties. Ceram. Int..

[32] Zhang X., Ni X., Li C., You B., Sun G. (2020). Co-gel strategy for preparing hierarchically porous silica/polyimide nanocomposite aerogel with thermal insulation and flame retardancy. J. Mater. Chem. A.

[33] Si Y., Sun J., Liu F., Shen H., Qi X., Xu Q., Zhou C., Liu Y., Fu Y. (2023). Rabbit-hair-like SiO_2_/PI composite nanofibers with super durability for thermal insulation. Compos. Part B Eng..

[34] Xi S., Wang Y., Zhang X., Cao K., Su J., Shen J., Wang X. (2023). Fire-resistant polyimide-silica aerogel composite aerogels with low shrinkage, low density and high hydrophobicity for aerospace applications. Polym. Test..

[35] Xue Q., Dong N., Fan P., Lin D., Tian G., Liu J., Wei J., Qi S., Wu D. (2024). Binary-network structured PI@SiO_2_ nanofibrous composite aerogels with temperature invariant superelasticity for thermal insulation. Chem. Eng. J..

[36] Wang W., Wang S., Li D., Yang J., Li H. (2025). Tailoring polyimide aerogel performance: The role of solid content in influencing microstructure, mechanical, thermal, and acoustic behaviors. RSC Adv..

[37] Kantor Z., Wu T., Zeng Z., Gaan S., Lehner S., Jovic M., Bonnin A., Pan Z., Mazrouei-Sebdani Z., Opris D.M. (2022). Heterogeneous silica-polyimide aerogel-in-aerogel nanocomposites. Chem. Eng. J..

[38] Xi S., Wang X., Liu T., Zhang Z., Zhang X., Shen J. (2021). Moisture-resistant and mechanically strong polyimide-polymethylsilsesquioxane hybrid aerogels with tunable microstructure. Macromol. Mater. Eng..

[39] Zhao L., Chen J., Pan D., Hou Y. (2023). Robust, fire-retardant, and water-resistant wood/polyimide composite aerogels with a hierarchical pore structure for thermal insulation. Gels.

[40] Hayase G., Kugimiya K., Ogawa M., Kodera Y., Kanamori K., Nakanishi K. (2014). The thermal conductivity of polymethylsilsesquioxane aerogels and xerogels with varied pore sizes for practical application as thermal superinsulators. J. Mater. Chem. A.

[41] Ergün M.E., Ergün H. (2023). Investigating the feasibility of guar gum-based foams for insulation applications using regression analysis. Dyna.

