# Supplementary material for: In Situ Polyimide Modified Polymethylsilsesquioxane Aerogels for Thermal Insulation
Source: Gels. 2026 Oct 4;12(10):898. doi: 10.3390/gels12100898 (PMC13704558; doi:10.3390/gels12100898)
Supplement: Supplementary file 1 [file gels-12-00898-s001.zip › Supplementary material.pdf]

This Supporting Information contains:

1. Supplementary Videos

2. Macroscopic Morphology of Si/PI composite aerogels

3. SEM images of Si/PI composite aerogels

4. N_2 adsorption-desorption isotherms and BJH pore-size distribution of Si/PI composite aerogels

5. TG curves of samples Si/PI-80

6. Environmental-stability evaluation of the Si/PI-80 composite aerogel

7. Comparison with reported composite aerogels

1.Supplementary Videos

Video S1

Video of Heating Process for Aerogel-insulated EPS Foam

Video S2

Video of Direct Heating on EPS Foam

Video S3

Video of the Combustion Process of EPS Foam

Video S4

Video of the Combustion Process of Si/PI-80 Aerogel

Video S5

The Burn-through Resistance Test of Sample Si/PI-80 Aerogel. This video has been sped up to 20X.

Video S6

Video of the Demonstration of Si/PI-80 Aerogel's Hydrophobic Properties

2.Macroscopic Morphology of Si/PI composite aerogels

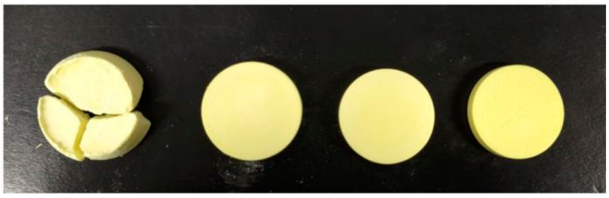

Figure S1. Macroscopic Morphology of Samples (from left to right: Si/PI-40, Si/PI-60, Si/PI-80, Si/PI-100).

3. SEM images of Si/PI composite aerogels

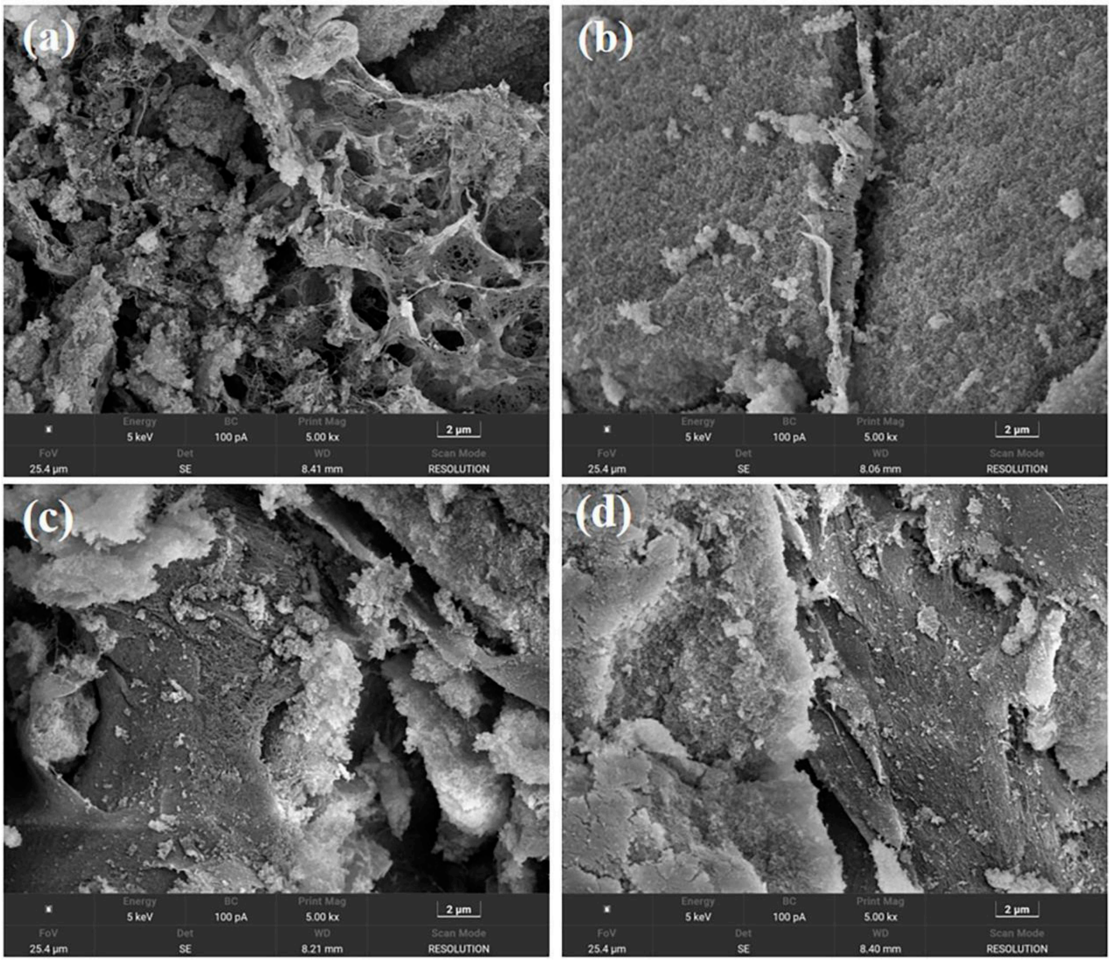

Figure S2. SEM image of samples (a) Si/PI-40 (b) Si/PI-60 (c) Si/PI-80 (d) Si/PI-100.

4. N_2 adsorption-desorption isotherms and BJH pore-size distribution of Si/PI composite aerogels

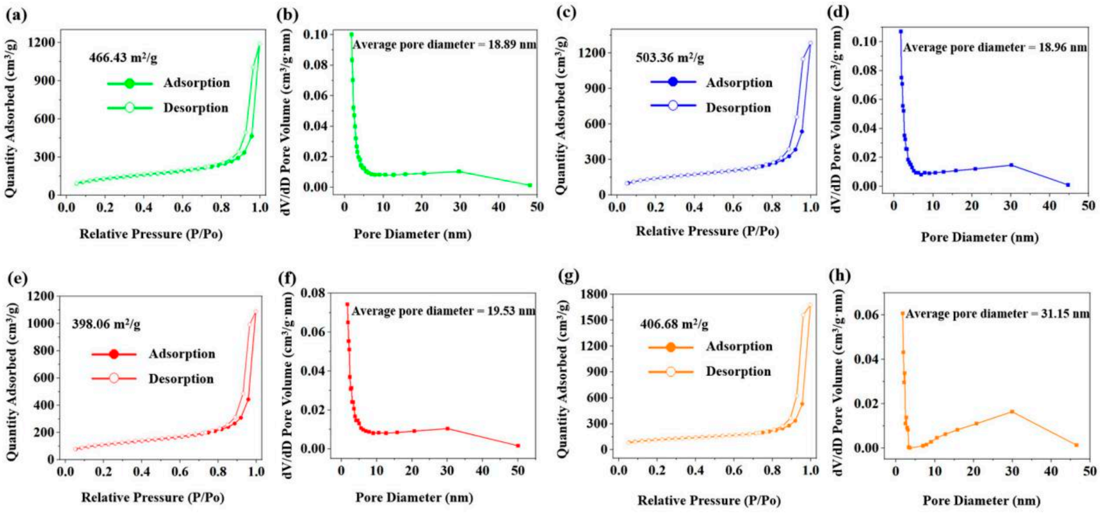

Figure S3. N_2 adsorption-desorption isotherms and BJH pore-size distribution of Si/PI composite aerogels. (a,b) Si/PI-40; (c,d) Si/PI-60; (e,f) Si/PI-80; (g,h) Si/PI-100. Note: Panels (a), (c), (e), (g) show N_2 adsorption-desorption isotherms; panels (b), (d), (f), (h) show corresponding BJH pore-size distributions.

5. TG curves of samples Si/PI-80

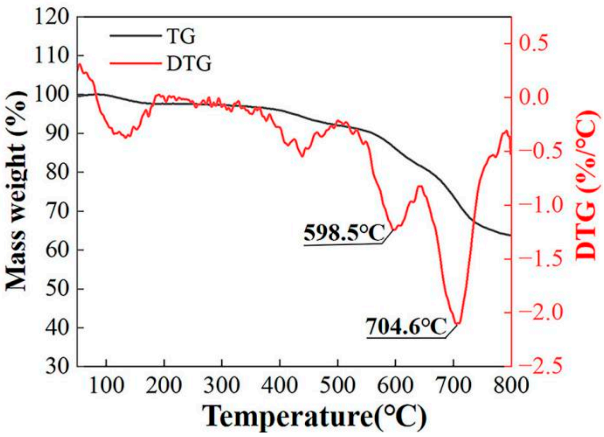

Figure S4. TG curves of samples Si/PI-80 (measured under ambient air conditions).

6. Environmental-stability evaluation of the Si/PI-80 composite aerogel

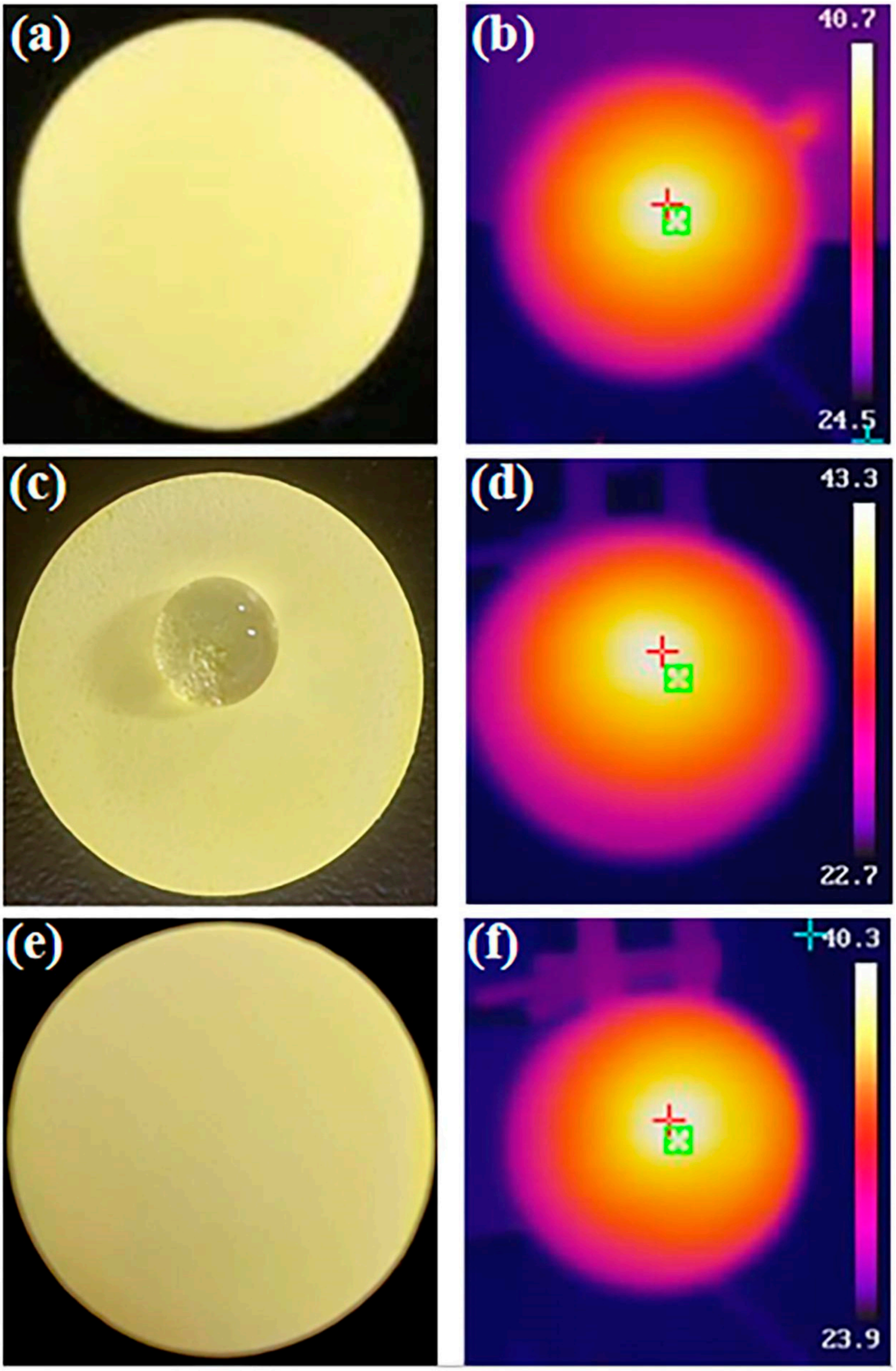

Figure S5. Environmental-stability evaluation of the Si/PI-80 composite aerogel. (a, b) Digital photograph of the pristine Si/PI-80 sample and its thermal-infrared image after direct contact with a point heat source at 110 ± 1 °C for 10 min; (c, d) Digital photograph

and thermal-infrared image of Si/PI-80 after 120 h exposure in a sealed saturated-vapor atmosphere at room temperature (~24 ℃); (e, f) Digital photograph and thermal-infrared image of the humidity-pre-treated Si/PI-80 after further thermal treatment at 250 ℃ for 48 h.

7. Comparison with reported composite aerogels

Table S1. Comparison of physicochemical properties of the Si/PI composite aerogel with previously reported polyimide-based aerogels.

Samples	Density ($mg\cdot cm^{-3}$)	Thermal conductivity ($W\cdot m^{-1}\cdot K^{-1}$) (25 ℃)	Mechanical Properties	Water contact angle (°)	Flame-retardant performance	Ref.
Si/PI-4	182	0.036	2.10 MPa @30 % strain	124	Resist 1300 ℃ butane-torch flame for 20 min, self-extinguishing	[6]
SiO_2/PI-3	Not explicitly provided	0.0311	Compressive modulus 1.96 MPa (0-5 % elastic region, large-strain	125	1300 ℃ butane-torch flame test only for 10 min	[32]

Samples	Density (mg·cm^{-3})	Thermal conductivity (W·m^{-1}·K^{-1}) (25 °C)	Mechanical Properties	Water contact angle (°)	Flame-retardant performance	Ref.
			strength not reported)			
PI-I40	73	0.0247	1.80 MPa (80 % strain)	141	Withstands 1300 °C butane-torch flame only for 1 min	[34]
Pyr_40_3	150	0.0175	Compressive modulus 1.8 MPa	>150	Only hot-plate thermal-stability test	[37]
Si/PI-80	182	0.03497	Compressive modulus 7.52 MPa	140.3	After continuous ablation by alcohol-lamp outer flame for 5 min: only slight carbonization on front surface	This work
